# Nutritional upgrade of olive mill stone waste, walnut shell and their mixtures by applying solid state fermentation initiated by *Pleurotus ostreatus*

**DOI:** 10.1038/s41598-024-64470-1

**Published:** 2024-06-11

**Authors:** Dimitrios Arapoglou, Christos Eliopoulos, Giorgos Markou, Ioanna Langousi, Georgia Saxami, Serkos A. Haroutounian

**Affiliations:** 1 Institute of Technology of Agricultural Products, Hellenic Agricultural Organization - DIMITRA (ELGO - DIMITRA), Sof. Venizelou 1, 14123 Athens, Greece; 2 Laboratory Nutritional Physiology and Feeding, Department of Animal Science, Agricultural University of Athens, Iera Odos 75, 11855 Athens, Greece

**Keywords:** Olive mill waste, *Pleurotus ostreatus*, Solid state fermentation, Protein enrichment, Sustainable management, Agro-industrial by-products’ valorization, Environmental biotechnology, Environmental impact

## Abstract

Present study concerns the transformation of the agro-industrial by-products olive mill stone waste (OMSW) and walnut shell (WS) to a protein-enriched animal feedstuff utilizing the solid state fermentation (SSF) technique. For this purpose, various mixtures of these by-products were exploited as substrates of the SSF process which was initiated by the *P. ostreatus* fungus. The respective results indicated that the substrate consisted of 80% WS and 20% OMSW afforded the product with the highest increase in protein content, which accounted the 7.57% of its mass (69.35% increase). In addition, a 26.13% reduction of lignin content was observed, while the most profound effect was observed for their 1,3–1,6 β-glucans profile, which was increased by 3-folds reaching the 6.94% of substrate’s mass. These results are indicative of the OMSW and WS mixtures potential to act as efficient substrate for the development of novel proteinaceous animal feed supplements using the SSF procedure. Study herein contributes to the reintegration of the agro-industrial by-products aiming to confront the problem of proteinaceous animal feed scarcity and reduce in parallel the environmental footprint of the agro-industrial processes within the context of circular economy.

## Introduction

World’s population is expected to face an unprecedented increase by 2050. To fulfil the projected boom of nutritional demand, the global food production must be amplified by 60%. This is expecting to generate huge amounts of by-products, derived mostly by the agro-food industries^1^. According to a Food and Agriculture Organization (FAO) report, nowadays the one-third of the manufactured products for human consumption (approximately 1.3 billion tons/year) is ending-up as waste^2^. In particular, it is estimated that only within the European Union territory, annually 88 Mtons of by-products are generated^3^. Part of this volume, accounting 9 Mtons, is derived by the primary sector, while an additional volume of 17 Mtons is generated by the agro-industrial processes^4^. Thus, there is an emerge for the development of efficient methods for their environmentally sound management. The latter comprises a significant global challenge, since the inadequate disposal of the aforementioned wastes constitutes a serious environmental burden which greatly contributes to groundwater pollution and increases the greenhouse gases emissions contributing to the climate change^5,6^. On the other hand, it must be noted that these by-products have a rich nutritional composition, since they contain a broad variety of nutrients, minerals, sugars, proteins, and they are characterized as lignocellulosic materials due to their increased content in cellulose, hemicellulose and lignin.

Because of their physicochemical composition, these by-products exhibit the potential to be exploited for a plethora of industrial applications^5–7^. In specific, olive tree is the prevailing orchard cultivation in the Mediterranean basin countries, with Spain, Italy, Greece, Portugal being the main producers. Olive oil is the main product of olive cultivation, which is extracted from olives by centrifugation utilizing two- or three-phase centrifuge systems^8,9^. The olive oil production is closely connected with the co-production of a liquid by-product termed as Olive Mill Waste Water (OMWW)^8,10^. The utilization of a three-phase centrifuge system results in the generation of OMSW as a heterogeneous biomass containing an increased amount of moisture along with the non-extracted oil. On the contrary, the two-phase system affords as by-product a liquid phase composed mainly by a black semi-solid component. The currently applied practice of disposing these by-products into open fields is considered as a significant environmental threat, since they are characterized by a high organic content and increased concentration of phenolic compounds^8–10^. Currently, the most environmentally acceptable method for their management refers to the isolation of their phenolic compounds, as mixtures and/or as individual compounds, aiming to their incorporation as potent antioxidants in nature based cosmetics and/or OTC (Over The Counter) pharmaceuticals ^10,11^.

Walnuts are derived from *Juglans regia* L, widely known as walnut tree. This plant is mainly cultivated in central Asia and in various areas of Europe, USA, western China, Iran and Afghanistan. In 2019 the global production of walnuts reached the 3.7 Mtons, highlighting this crop as the second most significant nut product^12^. China is the larger walnut producer, with an annual production volume reaching the 1.06 Mtons, followed by the USA and Iran which respectively produced 600 and 405 tons. Walnut shell (WS) constitutes the major by-product of its cultivation, representing the 67% of crop’s total weight^13^. WS is a lignocellulosic material mainly composed by lignin, hemicellulose and cellulose in amounts depending on crop’s source^14,15^.

These by-products display the common characteristic of poor nutritional value for utilization as animal feed. This constitutes a significant drawback for their incorporation into livestock rations, emerging the necessity to develop effective strategies for the enhancement of their nutritional quality^16^. In this respect, their treatment with white rot fungus comprises an intriguing case, since the application of this approach address the issue of low protein and the presence of high fiber substances. For this purpose, the edible white rot fungus *Pleurotus ostreatus* constitutes a suitable lignocellulosic degradation agent because of its ability to act with its hydrolytic and oxidative enzymes. Hydrolytic enzymes are especially effective in lignin’s modification, whereas the secretion of oxidative enzymes is more efficient in cellulose’s and hemilcellulose’s degradation^17^. Thus, the *P. ostreatus* fungus was selected as a potent lignocellulosic degradation agent for the successful application of Solid-State Fermentation (SSF)^18^, since displays the potential to enhance the nutritional profile of the investigated substrates by increasing their protein and amino acids content. For example, according to Heidari et al.^19^ report, the initiated by *P. ostreatus* SSF of canola meal increased its methionine, isoleucine, phenylalanine and tyrosine content. They also determined that methionine’s increase had a very positive effect on animals’ health and growth.

The SSF comprises a modern process that proceeds in a humid environment and is consistent with the efficient management of the solid by-products of agro-industrial process, which are easily assimilable by microorganisms. The SSF technique has the advantage to proceed in the absence of organic solvents, requires limited operational costs and provides high-quality extracts^20^. Hence, SSF process is considered as one of the most environmentally acceptable biotechnological procedures that can be used in a plethora of applications, such as the production of bioactive substances and metabolites, as well as the transformation of organic wastes, such as the agricultural by-products, the municipal or domestic food remaining, along with side-products generated from industrial processes, into high-added value products^21^.

Main objective of the present study concerns the nutritional upgrade of OMSW and WS by-products and their mixtures, in respect to their protein, nutrient and β-glucans content. For this purpose, we exploited the operational conditions and outcomes as well as the various aspects of the application of this initiated by *P. ostreatus* environmentally sound process. Final goal is to develop and establish a novel pathway for their transformation into novel proteinaceous animal feed.

## Materials and methods

### Materials and microorganisms

The OMSW and WS raw materials and their mixtures prepared by adding 20%, 40%, 60%, and 80% w/w WS to OMSW were utilized as substrates for the successful fermentation with *P. ostreatus*. After drying, these substrates were milled to a 2 mm particle size^22–24^. After hydration, a 300 g portion of each investigated substrate was placed into a closed test tube of final volume 750 mL and sterilized in autoclave by applying a temperature of 121 °C for 15 min. Their sufficient moisture content was regulated by the addition of tap water that was refreshed daily and preserved at 50% in order to assure the *P. ostreatus’* growth.

As inoculum we have utilized the provided by Fungi SEM, La Rioja, Spain commercial product of the *P. ostreatus* strain in solid form, "White 2000 P67 LOTTO 1551 MN 01827", which was stored in a refrigerator (4 °C) until utilization.

### Inoculation and solid-state fermentation procedures

A vertical laminar flow chamber was utilized for the inoculation experiments. Briefly, *P. ostreatus* strain was added on the surface of the investigated samples at a 3% w/w ratio and the mixture was transferred into an incubator keeping the temperature around 25 °C. The incubation was carried out in the dark and lasted for 14 days. All experiments were performed in triplicate.

### Analytical methods

The parameters of moisture, proteins and contained ash were determined by applying the respective AOAC methods^25^. The determination of total and reducing sugars content was carried out on the aqueous extracts of samples examined, in accordance with the methods reported by Dubois et al.^26^ and Miller^27^, respectively. The acid-detergent fiber (ADF) method was used for the evaluation of cellulose and lignin concentrations^25^. Finally, the content of 1,3–1,6 β-glucans was assessed by utilizing the enzymatic assay kit obtained from MEGAZYME (β-Glucan Assay Kit Yeast & Mushroom, Megazyme Product code: K-YBGL). All determinations were performed on dry samples and the obtained results are expressed as g/100 g.

### Statistical analysis

All experiments were carried out in triplicate and the respective results have been presented as mean ± standard deviation (± SD). Data normality was evaluated by applying the Shapiro–Wilk test. Group differences were examined by applying a paired t-test, with significance set at *p* < *0.05*.

## Results

Table 1 contains the physicochemical results of the investigated substrates of OMSW, WS and their mixtures concerning their moisture content, total and reducing soluble sugars as well as ash’s concentration.

**Table 1:** Determination of the physicochemical properties of substrates exploited.

Ratios (%w/w)	Moisture (%)		Total soluble sugars-TSS (%)		Reducing soluble sugars-RSS (%)		Ash (%)	
OMSW–WS	*Day* *0*	*Day* *14*	*Day* *0*	*Day* *14*	*Day* *0*	*Day* *14*	*Day* *0*	*Day* *14*
100 OMSW	48.84 ± 0.02	49.96 ± 0.51	1.02 ± 0.03	1.10 ± 0.05	0.48 ± 0.09	0.50 ± 0.07	4.72 ± 0.17	4.96 ± 0.04
80–20	48.12 ± 0.13	48.98 ± 0.55	0.63 ± 0.03^c^	1.09 ± 0.04^d^	0.23 ± 0.04^k^	0.48 ± 0.06^l^	3.50 ± 0.03	3.79 ± 0.67
60–40	51.49 ± 0.55	52.16 ± 0.61	0.67 ± 0.05	0.77 ± 0.03	0.37 ± 0.07^m^	0.15 ± 0.03^n^	2.21 ± 0.50^s^	3.43 ± 0.19^t^
40–60	50.24 ± 0.67	52.12 ± 0.40	0.45 ± 0.03^e^	0.89 ± 0.04^f^	0.13 ± 0.02	0.18 ± 0.02	3.05 ± 0.23	3.08 ± 0.06
20–80	48.26 ± 0.16	52.30 ± 0.30	0.47 ± 0.02^g^	1.12 ± 0.05^h^	0.12 ± 0.03^o^	0.33 ± 0.01^p^	2.73 ± 0.12	3.22 ± 0.30
100 WS	53.84 ± 1.01^a^	60.40 ± 0.16^b^	0.33 ± 0.01^i^	1.29 ± 0.04^j^	0.13 ± 0.01^q^	0.48 ± 0.02^r^	2.23 ± 0.05	2.87 ± 0.19

Statistical analysis was conducted separately for each evaluated attribute and sample among Days 0 and 14. Different superscripts display the presence of statistical significance (*p* < *0.05*).

The determined values for moisture content of all investigated substrates were almost identical, revealing a slight increase between Days 0 and 14. More specifically, the substrate consisted of 100% w/w OMSW underwent a slight increment of 2.29% at the end of the process. The moisture content of substrate prepared with the addition of 20% w/w WS remained practically unaffected ranging from 48.12% (Day 0) to 48.98% (Day 14), whereas substrates obtained with 40%, 60% and 80%w/w addition of WS displayed an increased to their moisture content by 1.30, 3.74% and 8.37%, respectively. Finally, for substrate composed by 100% w/w WS a statistically significant increment of moisture content by 12.18% (*p* < 0.05) was recoded at the end of the procedure. On the contrary, the amount of total soluble sugars (TSS) were increased for all investigated substrates. In specific, substrates prepared by the addition of 20% w/w WS, its TSS amount displayed a statistically significant (*p* < 0.05) increment of 73.02%, whereas for sample obtained with 40% w/w addition of WS the TSS amount was slightly increased at the end of the process. The TSS amount determined for substrate obtained with the 60% w/w addition of WS exhibited a statistically significant (*p* < 0.05) increase, which was recorded as a double amount on Day 14. Similar behavior was observed for substrate prepared by the addition 80% w/w of WS, since a TSS amount exceeding the two-folds increase was detected, while the respective substrate containing only OMSW demonstrated the greatest statistically significant increment by 4-folds (*p* < 0.05). Respectively, a similar behavior was also observed for their reducing soluble sugars (RSS) content, which underwent a statistically significant increase for almost all investigated substrates. In particular, for the OMSW substrate the amount of RSS remained practically unchanged, whereas the addition of 20% w/w WS resulted in the RSS doubling. The 60% w/w WS addition increased by 38% the RSS content, while the substrates obtained by 80% and 20% w/w addition of WS led to a statistically significant increase (*p* < 0.05) of RSS that exceeded the 2-folds, the highest RSS increase was determined for the substrate containing 100% w/w of WS which displayed the highest RSS content that exceeded the 3-folds increment (*p* < 0.05). It must be noted that the utilization of OMSW-WS (60–40% w/w) substrate resulted in the RRS volume reduction by 59.45% (*p* < 0.05). Finally, their ash content was increased in all cases, with the substrate obtained by the addition of 40% w/w of WS displaying a 55.20% increase (*p* < 0.05) to reach the 3.43% on Day 14.

Table 2 depicts the crude fiber substances (CFS) content of the investigated substrates determined during mycelium growth from Day 0 until Day 14 when the SSF procedure was terminated. More specifically, the CFS levels were varied among the investigated substrates with substrate composed by 100% w/w OMSW as well those containing 40% and 60% w/w WS recording an increase of 11.04%, 7.69 and 3.81. On the contrary, for substrates prepared by 20% and 80% w/w of WS a reduction of 8.39% and 23.67% was recorded (*p* < 0.05), whilst the substrate containing100% w/w of WS displayed the highest statistically significant reduction of 27.76% (*p* ≤ 0.05) at Day 14. For cellulose, the determined amounts at the end of fermentation displayed a similar behavior with substrates containing 20% and 60% w/w of WS presenting a statistically significant (*p* < 0.05) increment by 26.82% and 26.51% respectively, while the 100% w/w OMSW substrate recorded the highest statistically significant increase of 75.2% (*p* ≤ 0.05). Finally, the 100% w/w of WS substrate exhibited the most significant reduction by 9.05%. In respect the lignin content, a different pattern was recorded since a reduction for all studied substrates was revealed. In particular, the substrates containing 100% OMSW and 20%, 60% and 80% w/w of WS displayed significant reductions (*p* < 0.05) of their lignin contents, with the 20–80% w/w OMSW-WS ratio substrate revealing the highest statistically significant reduction by 26.13% (*p* ≤ 0.05) between Days 0 and 14.

**Table 2:** Determination of fiber substances, Cellulose and Lignin presence in the investigated samples.

Ratios (%w/w)	Crude fiber substances-CFS (%)		Cellulose (%)		Lignin (%)	
OMSW–WS	*Day 0*	*Day 14*	*Day 0*	*Day14*	*Day 0*	*Day 14*
100 OMSW	39.31 ± 0.22	43.65 ± 0.33	18.71 ± 0.43^e^	32.78 ± 0.06^f^	35.26 ± 1.45^k^	27.08 ± 1.37^l^
80–20	49.67 ± 1.08	45.50 ± 0.32	27.06 ± 1.39^g^	34.32 ± 0.37^h^	33.27 ± 1.09^m^	26.16 ± 0.50^n^
60–40	48.09 ± 0.89	51.79 ± 1.09	30.95 ± 1.31	33.18 ± 1.04	30.61 ± 1.68	29.41 ± 1.03
40–60	53.94 ± 1.26	56.00 ± 0.20	27.76 ± 1.87^i^	35.12 ± 1.42^j^	33.47 ± 1.01^o^	27.84 ± 0.65^p^
20–80	64.04 ± 1.20^a^	48.88 ± 1.42^b^	35.16 ± 1.34	32.81 ± 1.28	36.47 ± 1.44^q^	26.94 ± 1.46^r^
100 WS	70.20 ± 0.02^c^	50.71 ± 0.45^d^	40.42 ± 0.80	36.76 ± 1.10	31.40 ± 1.25	26.79 ± 1.63

Statistical analysis was conducted separately for each evaluated attribute and sample among Days 0 and 14. Different superscripts display the presence of statistical significance (*p* < *0.05*).

Finally, Table 3 summarizes the results of protein content and 1,3–1,6 β-glucans profile, revealing that both parameters were increased between Days 0 and 14 for all examined substrates. In specific, the amount of contained proteins increased during the SSF procedure with the substrates obtained with the addition of 20% w/w, 80%w/w and 100% of WS representing statistically significant (*p* ≤ 0.05) increase at Day 14, whereas the substrate composed by 100% w/w of WS presented the greatest increment exceeding two-folds, while the OMSW-WS mixture of 20–80% w/w ratio displayed the highest value. In respect the 1,3–1,6 β-glucans content, as indicated in Table 3, their amount at Day 14 was significantly increased (*p* ≤ 0.05) for almost all substrates investigated, with substrate obtained with OMSW-WS (20–80% w/w) ratio revealing the greatest statistically significant increase) and the highest content of 6.94%, which exceeded the 3-folds (*p* ≤ 0.05) increase.

**Table 3:** Determination of proteins and 1,3–1,6 β-glucans content in the examined substrates.

Ratios (%w/w)	Proteins (%)		1,3–1,6 β-glucans (%)	
OMSW–WS	*Day 0*	*Day 14*	*Day 0*	*Day 14*
100 OMSW	7.07 ± 0.50	7.18 ± 0.31	2.83 ± 0.14^g^	4.86 ± 0.32^h^
80–20	6.54 ± 0.03^a^	7.37 ± 0.12^b^	2.40 ± 0.23 ±	2.73 ± 0.11
60–40	6.01 ± 0.16	6.49 ± 0.63	1.79 ± 0.21^i^	4.02 ± 0.54^j^
40–60	4.94 ± 0.08	5.16 ± 0.29	2.25 ± 0.12^k^	5.46 ± 0.19^l^
20–80	4.47 ± 0.12^c^	7.57 ± 0.51^d^	2.09 ± 0.08^m^	6.94 ± 0.61^n^
100 WS	2.63 ± 0.18^e^	5.97 ± 0.13^f^	3.62 ± 0.11^o^	4.95 ± 0.47^p^

Statistical analysis was performed for each examined parameter and substrate individually between Days 0&14. Different superscripts indicate statistical significance (*p* < *0.05*).

## Discussion

The agro-industial by-products OMSW, WS and their mixtures were thoroughly investigated as substrates for the SSF with *P. ostreatus* fungus aiming to highlight the most suitable and efficient. Substrate’s efficacy is determined according to the nutritional profile of fermentation outcome, especially in respect to its protein content. As total incubation time was determined the period of 14 Days, which was considered as the most efficient period for the SSF performance since at that time *P. ostreatus* completely colonized the investigated substrates. Furthermore, the best results concerning all examined substrates were recorded on Day 14, especially in respect the observed significant increment of proteins and 1,3–1,6 β-glucans content with the substrate consisted of 20%OMSW-80%WS mixture being the most efficient.

Moisture comprises a significant parameter that greatly impacts the SSF process. Fan et al.^22^ have described that a moisture content of approximately 60% is considered as the ideal proportion for the achievement of best growth. Although a moisture content exceeding the optimum value is capable to facilitate the fungal growth and nutrients transfer, it is also noticeable that increased moisture levels have many negative effects on SSF implementation, since the produced water layer has the ability to overlay the substrate’s particles reducing the distance among them, limiting thus the air diffusion. Furthermore, the elevated moisture levels increase the risk of contamination since the nutritional content of generated substrates form an environment suitable for the growth of unfavorable microorganisms^23^. On the other hand, a low moisture in concentrations below the optimum levels mitigates nutrients’ solubility resulting in their insufficient absorption by fungi^22,23^. According to literature reports it is observed that moisture content fluctuates during the SSF process which depends on the nature of the examined materials^4,18^. Herein, throughout all experiments the moisture content was consistently preserved at approximately 60% for the substrate consisted by 100% w/w of WS since it ranged from 53.84 to 60.40% between Days 0 & 14, while for all other experiments it was determined around 50%.

The crude protein content constitutes the most important parameter for the evaluation of method’s efficacy, since the main focus of exploited bioconversion is to transform the substrate by-product into a protein-rich material. The results obtained after end of the initiated by *P. ostreatus* SSF revealed that the protein content was increased in all cases. According to Darwish et al.^28^ this increment may be associated with the potential accumulation of fungal biomass, whereas according to Akinfemi et al.^29^ crude proteins increase could be justified by the potential nitrogen’s absorption via aerobic fermentation. Moreover, Oseni and Akindahunsi^30^ claimed that the increased levels of crude proteins may be attributed to the secretion of specific protein-based extracellular enzymes capable to degrade the lignocellulosic materials in the SSF process. Finally, according to Terrasan and Carmona^31^ the elevated crude protein concentration may be associated with the microbial biomass colonization and growth on the respective substrates. In the present study, the obtained results revealed a substantial increase of crude protein levels which is in the line with these literature reports.

The degradation of lignocellulosic materials proceeds via a complex pathway. In this respect, the selection and utilization of suitable microorganisms such as fungi and especially basidiomycetes are crucial to assure the ability for successful implementation of SSF, since these microorganisms are also capable to act as degradation agents of their enzymatic systems content. In this respect, the white Rot fungi such as *P. ostreatus* are composed of two different groups of extracellular enzymatic systems, the hydrolytic and the oxidative. Its oxidative system secretes a specific extracellular lignin that is capable to modify the enzymes with laccase, lignin peroxidase and manganese peroxidases being considered as the most suitable for lignin’s degradation^29^. On the other hand, the hydrolytic system is mostly responsible for the degradation of cellulose and hemicellulose. Additionally, *Pleurotus* spp. can be characterized as selective degraders since they mainly modify lignin as compared to cellulose^3^.

The observed reduction of crude fiber substances (CFS) is presumably connected with the cellulase enzymatic activity that is secreted by the employed fungi^29^. Moreover, the involved microorganisms can act as energy source in order to fulfil their biological activities, justifying the observed reduction^30^. Thus, the amount of CFS was elevated in some substrates an observation that can be rationalized considering the parallel increment of cellulose or hemicellulose in the respective substrates. Another possible cause for the observed increase of CFS’ presence is connected with the activity of involved microorganisms that are capable to convert the examined substrate’s molecules into assimilable molecules used for the fulfilment of their biological activities. Such molecule is peptidoglycan, a polysaccharide that constitutes the major ingredient of bacterial wall. At the end of SSF process, the participating microorganisms are not separated from the biomass, resulting in the possible increment of CFS’s content due to their conversion to peptidoglycan^32,33^.

The volume of cellulose content presented a different behavioral pattern as compared to examined substrates, since for most substrates the respective amount was increased, whereas in two of the studied substrates was decreased. The increase of cellulose content can possibly be rationalized considering the different ratios of fiber fractions in studied substrates. It is also possible this increment may be resulted by the substrates ability to provide the utilized microorganism with essential nutrients such as N,P,K facilitating thus their degradation process^34^. On the other hand, this initiated by *P. ostreatus* procedure facilitates the more extensive degradation of lignin as compared to cellulose, resulting in a reduced content for all the investigated substrates. As we have already pointed out, *P. ostreatus* has been characterized as a selective degrader since exhibits the capability to degrade lignin more efficiently than cellulose. Therefore, the reduction of cellulose and lignin content can probably be explained by the efficacy of *P. ostreatus* to act as a degradation mean by secreting hydrolytic enzymes, facilitating the modification of cellulose and oxidative enzymes that promote the degradation of lignin. It must be noted however, that the specific procedure also assures the ability to improve substrates’ digestibility, emerging its potential for exploitation in the field of animal feed^3^.

Ash constitutes a significant physicochemical attribute as an indicator for the evaluation of their mineral content that contributes decisively into animal feeding rations^35^. At the end of the SSF process the ash content was increased for all studied substrates. This finding can be explained, considering the involvement of microorganisms to SSF procedure. Furthermore, according to Rajesh et al.^36^ the increase of contained ashes can be derived from the depleted organic matter utilized in the terms of SSF procedure.

The TSS and RSS concentrations displayed a somehow similar behavior, since at the end of SSF their content was increased for most substrates examined. This increment is probably associated with the excessive secretion of specific enzymes that are responsible for the degradation of lignocellulosic materials^35^. In some cases, the observed RSS reduction is presumably connected with their consumption by *P. ostreatus* since this material is comprises a valuable energy source for its growth.

Mushrooms and more specifically, *P. ostreatus* are highlighted for their rich nutritional content since they are composed by a plethora of bioactive compounds, including β-glucans. The latter are known to possess beneficial effects on human and animal health, acting as antitumor and immune-stimulating agents^37^. Chiozi et al.^38^ reported the incorporation of β-glucans derived from mushrooms into hens’ and fishes’ dietary promoted their health and welfare. In specific, the consumption of β-glucans by hens enhanced their immune system and improved the quality of the produced eggs, whilst the immune system of fishes was ameliorated and their lysozyme activity was enhanced. Herein the presence of 1,3–1,6 β-glucans was elevated at Day 14 in all investigated samples with substrate prepared by the addition of 80% w/w of WS to OMSW displaying the highest value of 6.94%. The observed increase of 1,3–1,6 β-glucans content of all examined substrates can be rationalized considering the potential of *P. ostreatus’* to exploit the nutrients contained in the respective substrates, for the production of organic compounds^35^. Thus, the obtained with SSF feedstuff constitutes a perfect protein source with a high 1,3–1,6 β-glucans content.

## Conclusions

The observed—during the last years—shortage of foodstuffs and feeds, is emerging the necessity for the discovery of novel, alternative sources of raw materials for their efficient production. In this respect, the valorization of agro-industrial by-products presents an intriguing case. It is evident that the adoption of strategies for the development of their efficient management and disposal practices is closely connected with beneficial impacts to the environment. In this respect, the production of economically feasible and environmentally sustainable components of animal feed comprises a high priority research goal. SSF procedure is a biotechnological process associated with the terms of circular economy. It must be noted that this is the first study that deals with the exploitation of OMSW, WS along with their mixtures. The present study highlighted the significance of agro-industrial by-products’ exploitation as novel raw materials targeting the reduction of their environmental impact. The respective results revealed that the substrate composed by an OMSW-WS (20–80%) w/w mixture upon application of an initiated by *P. ostreatus* SSF process*,* by bioconversion affords a high added value nutrient. In specific, the SSF process of this specific substrate provides at Day 14 a product with increased by 64.87% protein content, reduced by 26.13% lignin (the main antinutritional factor) and finally enhanced 1,3–1,6 β-glucans presence which reached the 6.94% at the end of the process. Outcome herein is indicative of this substrate’s potential for the production of animal feedstuff supplement. The final product is highlighted for its increased protein and 1,3–1,6 β-glucans content which are well known for their beneficial effects on animal health and welfare. Overall, SSF process demonstrates a new pathway for the management -via utilization- of agro-industrial by-products by improving their nutritional value in order to be transformed into potential feed additives in animals’ feedstuffs with a parallel economic impact, which also constitutes a solution of an environmental problem, within the context of circular economy.

## Data Availability

The datasets used and/or analysed during the current study available from the corresponding author on reasonable request.
